# Human Parasitic Diseases in Bulgaria in Between 2013-2014

**DOI:** 10.4274/balkanmedj.2017.0167

**Published:** 2018-01-20

**Authors:** Iskra Rainova, Rumen Harizanov, Iskren Kaftandjiev, Nina Tsvetkova, Ognyan Mikov, Eleonora Kaneva

**Affiliations:** 1 Department of Parasitology and Tropical Medicine, National Centre of Infectious and Parasitic Diseases, Sofia, Bulgaria

**Keywords:** Parasitic disease, prevalence, incidence, transmission, Bulgaria

## Abstract

**Background:**

In Bulgaria, more than 20 autochthonous human parasitic infections have been described and some of them are widespread. Over 50 imported protozoan and helminthic infections represent diagnostic and therapeutic challenges and pose epidemiological risks due to the possibility of local transmission.

**Aims:**

To establish the distribution of autochthonous and imported parasitic diseases among the population of the country over a 2-year period (2013-2014) and to evaluate their significance in the public health system.

**Study Design:**

Cross sectional study.

**Methods:**

We used the annual reports by regional health inspectorates and data from the National Reference Laboratory at the National Centre of Infectious and Parasitic Diseases on all individuals infected with parasitic diseases in the country. Prevalence was calculated for parasitic diseases with few or absent clinical manifestations (oligosymptomatic or asymptomatic infections). Incidence per 100.000 was calculated for diseases with an overt clinical picture or those that required hospitalisation and specialised medical interventions (e.g. surgery).

**Results:**

During the research period, parasitological studies were conducted on 1441.244 persons, and parasitic infections were diagnosed in 22.039 individuals. Distribution of various parasitic pathogens among the population displayed statistically significant differences in prevalence for some intestinal parasites (enterobiasis 0.81%, giardiasis 0.34% and blastocystosis 0.22%). For certain zoonotic diseases such as cystic echinococcosis (average incidence of 3.99 per 100.000) and trichinellosis (average incidence of 0.8 per 100.000), the incidence exceeds several times the annual incidence recorded in the European Union.

**Conclusion:**

Parasitic diseases still pose a substantial problem with social and medical impacts on the residents of our country. Improved efficiency regarding autochthonous and imported parasitic diseases is essential in providing the public health system the tools it needs to combat these diseases. Attention should be focused on the various imported vector-borne parasitic diseases (e.g. malaria and cutaneous leishmaniasis) for which the country is potentially endemic.

According to the World Health Organization (1), over 3 billion people around the world suffer from one or more parasitic diseases, and in most tropical or subtropical countries, they are widespread and a leading cause for morbidity and mortality in the population. In Bulgaria, more than 20 human parasitic infections with autochthonous distribution have been described and some of them are widespread: enterobiasis, hydatid disease, and trichinellosis (2). Growing relationships with tropical and subtropical countries increase the risk of inadvertently importing tropical parasitic diseases. Over 50 imported protozoan and helminthic infections represent diagnostic and therapeutic challenges and pose epidemiological risk due to the possibility of local transmission. More than 2 million Bulgarians travel annually across all regions of the world (3). The geopolitical changes in recent years have led to strong migratory pressure on European countries (including Bulgaria) due to migrants coming from the Middle East and North and sub-Saharan Africa. This mass movement of people increases the risk of the introduction and spread of parasitic pathology that currently has no local distribution.

The aim of this study was to conduct a retrospective epidemiological analysis on the prevalence of autochthonous and imported human parasitic diseases in Bulgaria for a period of 2 years (2013-2014) and to evaluate their significance on the public health system in the country.

## MATERIALS AND METHODS

The use of these data was consistent with the regulations of the National Centre of Infectious and Parasitic Diseases (NCIPD), Sofia, Bulgaria (Approval number: 1000-107/20.02.17)

### Surveillance system

Under current regulations, 11 parasitic diseases are subject to mandatory reporting and registration: ascariasis, cryptosporidiosis, congenital toxoplasmosis, echinococcosis, giardiasis, hymenolepiasis, malaria, taeniasis, trichinellosis, trichuriasis and visceral leishmaniasis (4). Surveillance and control of all parasitic diseases is carried out in accordance with the Ministry of Health (MoH) regulation regarding ‘the diagnosis, prevention and control of local parasitic diseases’ (5). Data on diagnosed cases are provided to the respective administrative district by the Departments of Medical Parasitology and Epidemiology at the Regional Health Inspectorates (RHI). In the Department of Parasitology and Tropical Medicine at the NCIPD, the information is summarised, analysed and presented to the MoH.

### Patients and samples

According to the country’s legislation, the following groups of people undergo an annual mandatory check-up for intestinal parasites: children attending nurseries and kindergartens and the personnel working in them, the staff in public food services, persons working in the field of production and trade of food products, and migrants and refugees who arrived in Bulgaria.

Our survey includes all Bulgarian and foreign nationals residing in the country who were examined for parasitic diseases from January 2013 to December 2014.

### Parasitological diagnosis

In Bulgaria, parasitological diagnosis is carried out in 89 laboratories: 28 laboratories at RHIs and 61 independent medical diagnostic laboratories or laboratories in hospitals based in the 28 districts of the country. Routine and confirmatory diagnostic activities are carried out at the National Reference Laboratory (NRL) at NCIPD.

Stool specimens were examined for intestinal helminths and protozoa by various methods: direct wet smear, Lugol’s iodine, formalin-ether and other concentration techniques and culture of larval-stage nematodes. For detection of *Enterobius vermicularis* eggs, the transparent tape test was used. For intestinal protozoa, staining techniques, culture methods and rapid immunochromatographic tests were used. For blood and tissue parasites, the following techniques were used: staining for examination of blood and bone marrow smears, culture methods, detection of specific antibodies by ELISA, Western blot, rapid immunochromatographic tests and molecular analysis by polymerase chain reaction (PCR). Immunological diagnosis and PCR were performed with certified, CE-marked commercial kits (Table 1).

### Data collection

We used the annual reports by RHIs on all individuals infected with parasitic diseases in the country and data from the NRL at NCIPD. Data from the National Statistical Institute on the country’s population size was also collected and these data were used to calculate parameters such as prevalence and incidence. Prevalence was calculated for parasitic diseases with few or absent clinical manifestations (oligosymptomatic or asymptomatic infections), and incidence per 100.000 was calculated for diseases with overt clinical picture or requiring hospitalisation and highly specialised medical care (e.g. surgery).

## RESULTS

During the research period, parasitological examinations were conducted on 1441.244 persons (713.446 or 10.2% of the population in 2013 and 727.798 or 9.9% of the population in 2014). A total of 22.039 (1.53%) patients were diagnosed with parasitic diseases (n=10.310, 1.44% for 2013, n=11.729, 1.61% for 2014) (Table 2). Diagnosis was confirmed by microscopic methods in 68% of the cases and in 32% by immunological or molecular methods.

### Diseases with local transmission

**Figure 1:**
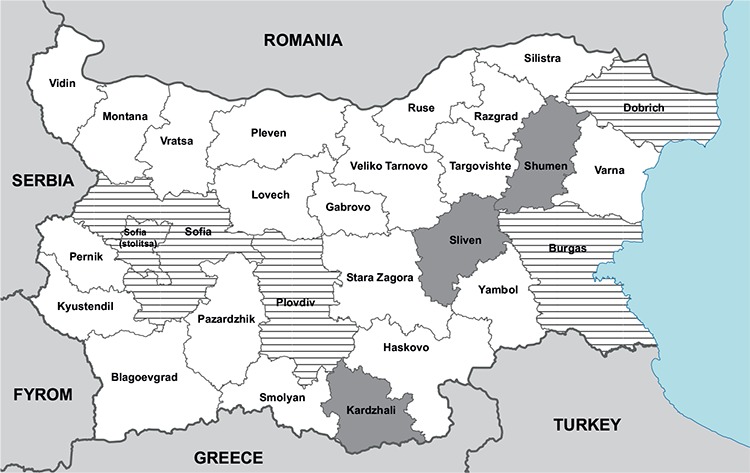
Administrative map of Bulgaria. The districts with higher incidence of cystic echinococcosis are marked with a solid fill. The areas with outbreaks of trichinellosis recorded during the studied period are marked with hatched lines.

**Cystic echinococcosis:** A total of 639 cases of hydatid disease were recorded. The relative share of primary cases was 90.85% (n=278 for 2013) and 92.69% (n=302 for 2014). Average incidence of hydatid disease in 2013 was 3.82 per 100.000, and 4.17 per 100.000 in 2014. The most affected groups were patients 20-29 years old (14%), 30-39 years old and 60-69 years old (13% both groups). Among children and adolescents (0-18 years), 113 (17.7%) cases of the disease (n=55 for 2013 and n=58 for 2014) were recorded. In 2014, 12 (20.69%) of the infected children were in the 0-4 year age group. Two deaths due to cystic hydatid disease (4-year-old boy and 48-year-old woman) were recorded. The highest incidence was registered in the region of Sliven with average incidence of 14 per 100.000 for 2013 and 2014, the region of Kardzhali with 12 per 100.000 and the region of Shumen with 9.8 per 100.000 (Figure 1).

**Trichinellosis**: Seven outbreaks (two in 2013 and five in 2014) were recorded in the country. A total of 261 persons consumed meat products contaminated with *Trichinella spiralis* larvae. Infection confirmed by laboratory tests (ELISA and Western blot) was established in 127 (48.66%) of the cases. Clinical symptoms developed in 123 (96.85%) patients and 4 (3.15%) remained asymptomatic. Out of the 127 people infected in both years, 60 (47.24%) were men, 46 (36.22%) were women and 21 (16.54%) were children and adolescents (6). In 4 of the outbreaks, the source of infection was meat from wild boar, 2 of the outbreaks could be attributed to the domestic pig, and in 1 outbreak, the source was mixed meat from wild boar and domestic pig. The average incidence of trichinellosis in the country was 0.8 per 100.000. Outbreaks were recorded in the regions of Sofia, Sofia-capital, Plovdiv, Dobrich and Burgas (Figure 1). The outbreaks associated with consumption of meat from wild boar were caused by *Trichinella britovi*, while those with meat from domestic pig by *T. spiralis*.

**Taeniasis:** During the study period, 59 cases of taeniasis caused by *Taenia saginata* were recorded. The incidence was 0.49 per 100.000 for 2013 and 0.32 per 100.000 for 2014. Autochthonous cases of intestinal *Taenia solium* infections were not registered, but 36 serologic tests for cysticercosis were conducted at NCIPD by clinical indications and positive results in ELISA were found in 2 patients with clinical manifestations of neurocysticercosis.

**Toxocariasis:** Four hundred sixty-six persons were examined for toxocariasis by serological tests using ELISA and Western blot, primarily due to peripheral blood eosinophilia and/or allergy symptoms. Positive test results were found in 90 patients (19.3%).

**Toxoplasmosis:** Tests for toxoplasmosis were conducted on pregnant women or women enrolled in programmes for *in vitro* fertilisation, patients with lymphadenitis, patients with vague febrile illness, patients with inflammation of the eye and immunocompromised patients with low CD4 cell counts. A total of 19.823 individuals were studied (n=10.368 in 2013 and n=9.455 in 2014). The presence of specific immunoglobulin G (IgG) antibodies was found in 3.962 (19.97%) of the tested persons, anti-Toxoplasma IgG and immunoglobulin M (IgM) antibodies in 177 (0.89%) patients, and 33 (0.17%) persons were positive for IgG, IgM and immunoglobulin A. IgG avidity was examined in 32 patients, and of these, 13 were pregnant women in different stages of gestation. Low avidity was confirmed in 3 of the pregnant women

**Visceral leishmaniasis:** Twenty-seven cases of visceral leishmaniasis caused by *Leishmania infantum* were recorded in the country. Twenty-five of them were autochthonous and 2 were imported from Portugal and Greece. Twelve of the cases were in children, and 15 were in adults. Established incidence for the period was 0.19 per 100.000.

**Soil-transmitted helminth infections:** Ascariasis and trichuriasis have local transmission in Bulgaria. A total of 1 170.966 persons (n=589.417 in 2013 and n=581.549 in 2014) were examined. Of them, 1139 persons were positive for ascariasis, with an average incidence of 7.84 per 100.000 for the period. Also, 193 cases of trichuriasis were recorded with an average incidence of 1.37 per 100.000 for the period.

### Communicable parasitic diseases

**Enterobiasis:** During the study period, 1056.805 persons (500.251 in 2013 and 556.554 in 2014) were examined. The average national prevalence for the period was 0.81% (0.75% in 2013 and 0.87 % in 2014). Annually, more than 90% of the children in childcare facilities were examined for enterobiasis. During the studied period, 353.106 children aged 1-7 years were residing in different types of childcare. The established average prevalence of 1.1% in this group was higher than the national average.

**Giardiasis:** A total of 1062.600 persons (positive n=3604) were tested for giardiasis, and the average prevalence was 0.34% (0.36% in 2013 and 0.31% in 2014).

**Hymenolepiasis:** A total of 911.922 people were examined for hymenolepiasis and 664 of them were infected. The average prevalence for the period was 0.07%.

### Other intestinal protozoan infections

For cryptosporidiosis, 841 people were tested. Three patients (0.36%) were positive and 1 of them was also HIV positive. For blastocystosis 1005.828 persons (501.800 in 2013, and 504.028 in 2014) were examined, with an average prevalence in the country of 0.22% (n=2212).

### Imported parasitic diseases

During the 2-year study period, 8.970 persons were examined, including 611 Bulgarian citizens (Table 3). According to the current legislation, all persons accommodated in refugee camps in Bulgaria must be tested for intestinal parasites and those coming from endemic countries should also be tested for malaria. This is carried out by RHIs working in each of the 28 districts of the country.

**Malaria:** Bulgaria is certified as being malaria-free since 1965. A total of 3.838 persons were tested for malaria, including 553 Bulgarian citizens. Eighteen cases of imported malaria were diagnosed: 8 in 2013 and 10 in 2014. Most of the diseased were Bulgarian citizens (n=14). *Plasmodium falciparum* was identified as the causative agent in 14 cases and *Plasmodium vivax* in 4.

**Intestinal parasitic diseases:** Prophylactic examinations for intestinal parasites were conducted in 6.147 foreign nationals who sought asylum in the country. The examinations found the presence of the following parasites: *Giardia intestinalis, Blastocystis hominis, Enterobius vermicularis, Hymenolepis nana, Ascaris lumbricoides, *and* Trichocephalus trichiurus* (Table 3).

**Other imported parasitic diseases:** Two cases of cutaneous leishmaniasis, 1 case of loiasis and 1 case of urogenital schistosomiasis were diagnosed during the period, all of which do not have autochthonous transmission in Bulgaria.

## DISCUSSION

### Parasitic infections with local transmission

During the research period, around 10% of the country’s population was examined for parasitic infections. Two of these, including cystic echinococcosis (CE) and trichinellosis, have important medical and public health implications. A disturbing fact is the high proportion of echinococcosis among children and adolescents (17.7%, n=113), which indicates a high transmission rate in the country (7,8,9). The incidence of cystic hydatid disease in the European Union for the reviewed period was 0.18 per 100.000 (10,11), but in Bulgaria the incidence is 30-40 times higher. Possible reasons for the high incidence of human CE in Bulgaria is the large number of free-ranging stray dogs and the breeding of yard and shepherd dogs in some rural areas that are not regularly dewormed which, in turn, leads to a high infection rate among domestic animals (7). In a study of the prevalence of echinococcosis among livestock at slaughter meat production houses, animals that were most affected were the sheep (6.4%), followed by cattle (4.3%), goats (0.9%) and pigs (less than 0.1%) (7,12).

After the political and economic changes, human trichinellosis became a re-emerging zoonosis in Bulgaria since 1991. Between 1990 and 2006, 145 trichinellosis outbreaks and 238 sporadic cases were recorded (13). In 2013-2014, 536 cases of human trichinellosis (10,11) were recorded in the EU, of which 130 (24.3%) were registered in Bulgaria. Accordingly, the incidence was 0.05 (2013) and 0.07 (2014) per 100.000 for the EU, and 0.82 and 0.83 per 100.000 for Bulgaria (6). Regarding the *Trichinella* spp. causing outbreaks in the country, some previous (14) and recent studies (6) confirmed the invariable presence of 2 species, *T. britovi* and *T. spiralis*.

In Bulgaria, toxocariasis is not a notifiable disease, but according to previous studies of patients with allergy and/or eosinophilia, its seroprevalence ranges between 12.25% and 14.01%, (15,16,17,18). Our present survey of patients with allergy and/or eosinophilia established a seroprevalence of 19.3%, while earlier research (2005-2008) conducted among healthy blood donors (n=350) found a seroprevalence of 3.4% (19). Based on the aforementioned data, it can be approximated that the average prevalence of toxocariasis among persons with allergy and/or eosinophilia is 15.09%, which is approximately 5 times more than healthy people.

Although our study was not a screening of the Bulgarian population for toxoplasmosis, the seroprevalence values we found were similar to the those established by previous studies in Bulgaria (24.1% for 2011 and 20% for 2012) (18), and is slightly lower than values found in a 2004 Greek study that established seroprevalence among the general population as 24.1% (20). According to some authors, approximately 25%-30% of the world human population is infected by *Toxoplasma* spp. (21). Actually, the prevalence varies wildly between countries (from 10% to 80%) and often within a given country or between different communities in the same region (22). Over the 2013-2014 time period, primary toxoplasmosis was diagnosed in 13 pregnant women at NCIPD. Three of them had low IgG avidity. In pregnant women, it is not always easy to date the infection versus the time of conception, but a high avidity result in early pregnancy can generally help rule out infection in pregnancy in most cases of positive specific IgM.

Soil-transmitted helminth infections (ascariasis and trichuriasis) can be defined as ‘habitual’ for the country, because cases among the population are recorded every year at approximately the same level. Analysis of the data has shown that in recent years the total number of people infected with *A. lumbricoides *varies between 500 and 700 per year and for people infected with *T. trichiurus*, about 100 per year. Regions with the greatest number of ascariasis cases are in the southern part of the country where the climate is more suitable for parasite transmission. Trichuriasis is registered more often among people living in social institutions (for children deprived of parental care and adults in mental care facilities) (23). Ancylostomiasis is not endemic to the country and is without local transmission.

The communicable parasitic diseases are of particular health importance for Bulgaria because they affect mostly children in organised groups. Of these diseases, enterobiasis is still the most common and its prevalence levels remain high (24). Although the prevalence of giardiasis shows a declining trend, it retains its medical importance, while cases of hymenolepiasis are rarely recorded.

Cases of cryptosporidiosis in people are seldom diagnosed, although a pilot study employing immunofluorescence and PCR techniques proved the presence of *Cryptosporidium* spp. oocysts in various sources of drinking water (25). The low incidence of cryptosporidiosis among humans in the country is probably due to the relatively mild clinical symptoms in immunocompetent persons and to the relatively low number of HIV-positive persons (n=2046) (26).

Since 1989, autochthonous cases of visceral leishmaniasis caused by *Leishmania infantum* are annually registered in Bulgaria. By 2012, 122 cases of the disease were recorded, of which 118 were indigenous and 4 imported cases were from leishmaniasis-endemic European countries (Italy and Spain) (27). During this study, most of the autochthonous cases of visceral leishmaniasis were recorded in 2 municipalities (Petrich and Parvomay) located in southern Bulgaria, and 2 cases were imported from Greece and Portugal.

### Imported parasitic diseases

Over the course of our study, we observed imported parasitic diseases such as malaria, cutaneous leishmaniasis, urogenital schistosomiasis and loiasis, none of which had local transmission in the country. The rest of the imported cases were intestinal parasitic diseases such as soil-transmitted helminth infections (ascariasis and trichuriasis), communicable parasitic diseases (enterobiasis, hymenolepiasis, giardiasis, and blastocystosis) and visceral leishmaniasis, all of which have local distribution. The gathering of large groups of migrants with such parasitic infections in endemic areas may lead to formation of new foci of transmission or to an increase in the intensity of the pre-existing ones. It seems that the import of parasitic diseases from endemic regions will continue in the coming years. This influx is associated with expanding trade, political and economic relations, tourism and, unfortunately, mass migration of people. The country has favourable climate and fauna conditions for local distribution of a number of parasitic diseases. In 1995 a local outbreak of imported malaria that reported illegal immigrants from Afghanistan as a source was recorded (28). Similar cases have been reported in Greece between 2009 and 2013 with registered cases of tertian malaria with local transmission imported by migrants (29).

The local transmission of parasitic diseases directly depends on the effectiveness of the system for surveillance and control. Regardless of the improvement of living standards and tools for health education, parasitic diseases are a significant part of the overall pathology registered in Bulgaria. It is essential to constantly improve the efficiency of the public health system in order to deal with autochthonous parasitic diseases. It is also imperative to maintain a state of increased preparedness for action against tropical parasitic diseases that may be imported more often into the country in the coming years.

## Figures and Tables

**Table 1:**
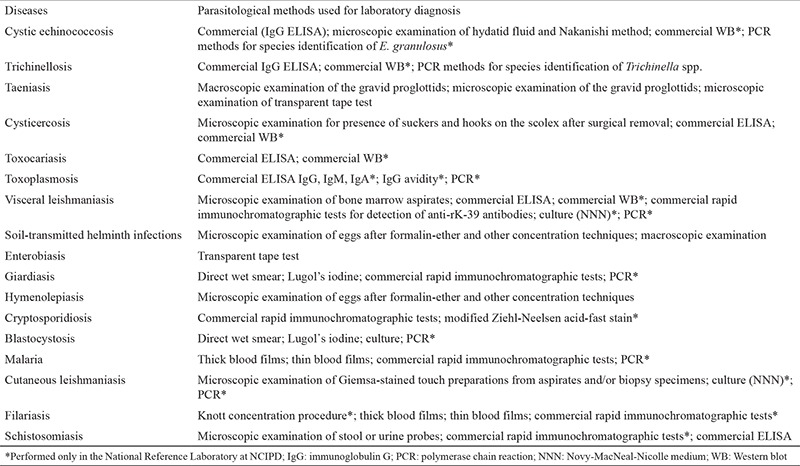
Methods used for laboratory diagnosis (according to the ‘Medical Parasitology’ medical standard issued by the Ministry of Health of the Republic of Bulgaria 23.12.2014)

**Table 2:**
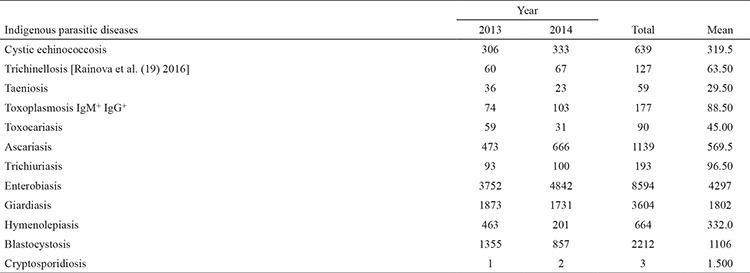
Data on registered autochthonous human parasitic diseases in Bulgaria 2013-2014

**Table 3:**
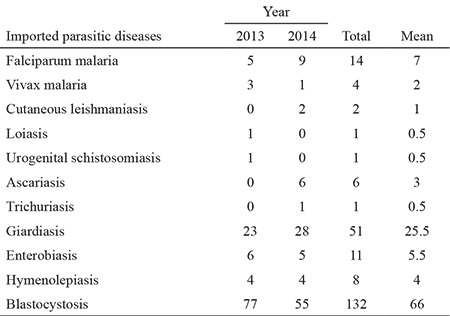
Data on recorded imported parasitic diseases in Bulgaria 2013-2014
